# Genome-wide association analysis identifies resistance loci for bacterial blight in a diverse collection of *indica* rice germplasm

**DOI:** 10.1371/journal.pone.0174598

**Published:** 2017-03-29

**Authors:** Fan Zhang, Zhi-Chao Wu, Ming-Ming Wang, Fan Zhang, Michael Dingkuhn, Jian-Long Xu, Yong-Li Zhou, Zhi-Kang Li

**Affiliations:** 1 Institute of Crop Sciences/National Key Facility for Crop Gene Resources and Genetic Improvement, Chinese Academy of Agricultural Sciences, Beijing, China; 2 Graduate School of Chinese Academy of Agricultural Sciences, Chinese Academy of Agricultural Sciences, Beijing, China; 3 Crop and Environmental Sciences Division, International Rice Research Institute, Los Baños, Laguna, Philippines; 4 Centre de Coopération Internationale en Recherche Agronomique pour le Développement (CIRAD), UMR AGAP, Montpellier, France; 5 Shenzhen Institute of Breeding and Innovation, Chinese Academy of Agricultural Sciences, Shenzhen, Guangdong, China; Fujian Agriculture and Forestry University, CHINA

## Abstract

Bacterial blight, which is caused by *Xanthomonas oryzae* pv. *oryzae* (*Xoo*), is one of the most devastating rice diseases worldwide. The development and use of disease-resistant cultivars have been the most effective strategy to control bacterial blight. Identifying the genes mediating bacterial blight resistance is a prerequisite for breeding cultivars with broad-spectrum and durable resistance. We herein describe a genome-wide association study involving 172 diverse *Oryza sativa* ssp. *indica* accessions to identify loci influencing the resistance to representative strains of six *Xoo* races. Twelve resistance loci containing 121 significantly associated signals were identified using 317,894 single nucleotide polymorphisms, which explained 13.3–59.9% of the variability in lesion length caused by *Xoo* races P1, P6, and P9a. Two hotspot regions (L11 and L12) were located within or nearby two cloned *R* genes (*xa25* and *Xa26*) and one fine-mapped *R* gene (*Xa4*). Our results confirmed the relatively high resolution of genome-wide association studies. Moreover, we detected novel significant associations on chromosomes 2, 3, and 6–10. Haplotype analyses of *xa25*, the *Xa26* paralog (*MRKc*; *LOC*_*Os11g47290*), and a *Xa4* candidate gene (*LOC*_*11g46870*) revealed differences in bacterial blight resistance among *indica* subgroups. These differences were responsible for the observed variations in lesion lengths resulting from infections by *Xoo* races P1 and P9a. Our findings may be relevant for future studies involving bacterial blight resistance gene cloning, and provide insights into the genetic basis for bacterial blight resistance in *indica* rice, which may be useful for knowledge-based crop improvement.

## Introduction

Bacterial blight, which is caused by *Xanthomonas oryzae* pv. *oryzae* (*Xoo*), is one of the most devastating diseases of cultivated rice (*Oryza sativa* L.) in tropical and temperate regions worldwide [1]. This disease is frequently prevalent in southern China and southeast Asia, resulting in heavy rice yield losses [2, 3]. Developing and deploying resistant cultivars carrying major resistance (*R*) genes has been the most effective approach for managing bacterial blight [4].

Bacterial blight is characterized by a high degree of race–cultivar specificity. Several sets of isotype races/pathotypes have been identified since the 1980s in the Philippines, China, and other countries in rice cultivars differing in susceptibility to bacterial blight [4–9]. Based on analyses of phenotypic responses to *Xoo* races and molecular mapping results for identified genes, 41 *R* genes (i.e., 29 dominant and 12 recessive genes) conferring resistance to bacterial blight have been registered in the Oryzabase database (http://www.shigen.nig.ac.jp/rice/oryzabase/gene/list). Most of these genes are derived from *Oryza sativa* ssp. *indica* cultivars, but seven genes are from six related wild species [10–12]. Additionally, some *R* genes or alleles have been generated by mutating cultivated rice lines [10, 13, 14], including the following nine isolated genes: *Xa1*, *xa5*, *xa13*, *Xa21*, *Xa23*, *xa25*, *Xa26/Xa3*, *Xa27*, and *xa41* [15–23]. Another nine genes have been fine-mapped (i.e., *Xa2*, *Xa4*, *Xa7*, *Xa22*, *Xa30*, *Xa33*, *Xa38*, *Xa39*, and *Xa40*) (http://www.shigen.nig.ac.jp/rice/oryzabase/gene/list). Moreover, quantitative trait loci (QTLs) for resistance to bacterial blight caused by different *Xoo* isolates have also been reported [24, 25].

Novel *R* genes and QTLs associated with bacterial blight resistance have been identified and used in breeding programs. However, the rapid loss of bacterial blight resistance in rice varieties carrying a single *R* gene remains a problem for breeders. This has been in part because the mechanisms mediating the pathogenesis of *Xoo* and the genetic basis for bacterial blight resistance in rice have not been fully characterized. To date, most of the studies on bacterial blight resistance have been based on a single resistant parent or bi-parental genetic mapping populations. Additionally, the genetic variability of bacterial blight resistance in rice accessions has yet to be addressed because only one or two parental lines have been used in previous studies. A genome-wide association study (GWAS) involving high-density single nucleotide polymorphisms (SNPs) based on next-generation sequencing may be useful for detecting genetic variants that can be directly applied to improve rice cultivars [26]. In this study, 172 global *indica* accessions were inoculated with representative strains of six *Xoo* races from China and the Philippines to evaluate their resistance reactions. We also conducted a GWAS of bacterial blight resistance based on the genotyping of 700,000 SNPs with a high-density rice array [27]. Our study objectives were to elucidate the genetic basis of bacterial blight resistance and identify loci related to bacterial blight resistance in *indica* rice lines, which may provide useful information for improved rice production.

## Materials and methods

### Plant materials and bacterial inoculations

We examined 172 *indica* rice accessions from 26 countries (S1 Table). To evaluate bacterial blight resistance, the seeds of all plant materials were sown in a seedling nursery, and 30-day-old seedlings were transplanted to the experimental farm at the Institute of Crop Sciences, Chinese Academy of Agricultural Sciences, Beijing, China. There were nine plants in each row (20 × 17 cm). The following six representative *Xoo* strains were used to artificially inoculate plants: GD1358 (race C5) and V (race GV) from China, and PXO61 (race P1), PXO340 (race P3c), PXO339 (race P9a), and PXO99 (race P6) from the Philippines. The bacterial races were grown on peptone sucrose agar medium at 30°C for 2 days, and each inoculum was prepared by suspending the bacterial mass in sterile water at a concentration of 10^8^ cells ml^−1^. Five central plants for each line were inoculated with each *Xoo* race (with two replicates) at the tillering stage (plant age: 65 days), and four or five of the uppermost leaves of each plant were inoculated with each *Xoo* race using the leaf-clipping method [28]. The lesion lengths (LLs) were measured on all inoculated leaves 3 weeks post-inoculation when lesions were obvious and stable. The average LL of one accession was calculated based on 15 of the longest lesions from five individual plants (i.e., three lesions per plant) for each replicate. The average LL of two replicates for each accession was used as the phenotype for the GWAS.

### Genotyping and population structure analysis

All 172 accessions were genotyped for 700,000 SNPs using a high-density rice array [27]. To avoid the influence of linked SNPs during the population structure analysis, we used the linkage disequilibrium (LD) pruning tool of PLINK [29] to obtain a subset of 45,311 independent SNPs with a minor allele frequency (MAF) > 5% and a missing data ratio (MDR) < 0.1 according to ‘indep-pairwise 50 10 0.5’. We used PHYLIP (version 3.6) [30] to construct an unrooted neighbor-joining tree with 100 bootstrap replicates. The genetic structure of the whole population was predicted with the ADMIXTURE program [31]. EIGENSOFT [32] was used to conduct principal components analysis (PCA) for estimating the number of subpopulations in the GWAS panel.

### Association mapping

A total of 279,266 SNPs with a MAF > 5% and MDR < 0.2 were filtered for association analyses of the whole panel. The GWAS was completed using a linear mixed effects model to determine the associations between each SNP and the phenotype (i.e., mean lesion length caused by *Xoo*). We applied the efficient mixed model analysis (EMMA) feature of the EMMA eXpedited (EMMAX) software [33]. We used the Balding–Nichols matrix based on genome-wide SNP data to develop the kinship matrix, which measured the genetic similarity between individuals. The first seven principal components explaining 80% of the genetic variation were used as covariates.

Based on 1000 permutation tests, the genome-wide significance thresholds at a significance level of 0.05 were *P* = 3.49 × 10^−7^, 1.82 × 10^−7^, 2.58 × 10^−6^, 3.15 × 10^−7^, 3.50 × 10^−7^, and 1.34 × 10^−7^ for *Xoo* races P1, P3c, P6, P9a, C5, and GV, respectively. The Manhattan and quantile-quantile plots for GWAS results were created using the R package qqman [34]. We obtained independent association signals using a published method [35]. Briefly, multiple SNPs exceeding the threshold in a 5-Mb region were clustered using an LD *r*^2^ value ≥ 0.25, and SNPs with the minimum *p* value in a cluster were considered as the lead SNPs. The pairwise LD *r*^2^ values were calculated with PLINK [29], and the R package LDheatmap [36] was used to draw the heatmap of pairwise LD. The SNPs spanning a region from 2 kb beyond the 3′ end of a gene to the 5′ end were concatenated as the haplotype. We excluded SNPs with no functional effect according to the haplotype analysis, and only used the haplotypes shared by at least five accessions. Phenotypic variations explained by multiple lead SNPs of association loci were estimated by stepwise regression using SAS [37].

### Analysis and annotation of significant signals

Synonymous and nonsynonymous SNPs and SNPs with large-effect changes were annotated based on the gene models of the annotated version of the Nipponbare reference genome (IRGSP 1.0) [38] using snpEff software (version 4.0) [39]. Enriched gene ontology terms were identified using the agriGO online tool [40]. We listed all significant SNPs located within genes and annotation information for the Nipponbare reference genome (IRGSP 1.0) [38].

## Results

### Population structure of rice accessions

We genotyped a diverse global collection of 172 *O*. *sativa* ssp. *indica* accessions (S1 Table) using a high-density rice array [27], resulting in 700,000 high-quality SNPs. A total of 45,311 independent SNPs with MAF > 5% and MDR < 0.1 were used for genetic structure analyses. The neighbor-joining tree developed using PHYLIP version 3.6 [30] with 100 bootstrap replicates and the ADMIXTURE results [31] revealed that the 172 accessions were grouped into three clusters (S1A and S1C Fig). A similar result was observed using PCA, in which most of the genetic variation in the accessions was explained by the first two principal components. When we plotted the first two components against each other, most accessions clustered in three groups with a few admixed accessions (S1B Fig). We then divided the 172 *indica* accessions into three subgroups, namely *ind-I* (78 accessions), *ind-II* (36 accessions), and *ind-III* (58 accessions) (S1 Table).

### Reactions of accessions to different *Xoo* races

**Fig 1:**
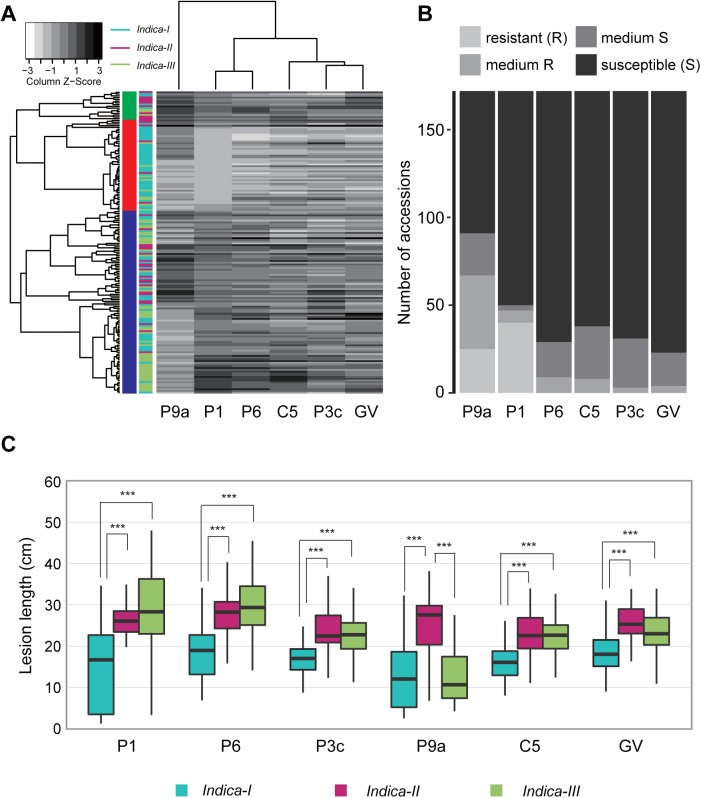
Susceptible and resistant reactions of a whole population and six subpopulations of 172 *indica* accessions inoculated with representative strains of six *Xanthomonas oryzae* pv. *oryzae* (*Xoo*) races from China and the Philippines. (A) Hierarchical cluster of accessions and races based on lesion length (LL). (B) Number of accessions in the following reactions to six *Xoo* races: resistant (LL < 5 cm), moderately resistant (5 cm ≤ LL < 10 cm), moderately susceptible (10 cm ≤ LL < 15 cm), and susceptible (LL ≥ 15 cm). (C) Boxplots for LLs following infections by six *Xoo* races in three *indica* subgroups divided by population structure analysis results. Box edges represent the 0.25 and 0.75 quantiles with median values indicated by bold lines. ‘***’ refers to a significant difference in the average LLs among the *indica* subgroups (*p* < 0.001). We used the following *Xoo* races: C5 (strain GD1358) and GV (strain V) from China, and P1 (strain PXO61), P3c (strain PXO340), P9a (strain PXO339), and P6 (strain PXO99) from the Philippines.

All accessions were inoculated with six *Xoo* races at the primary tillering stage (S1 Table). Based on the LL values calculated using the unweighted pair-group method with arithmetic mean, the six bacterial races were divided into three groups. One group consisted of race P9a, another group comprised races P1 and P6, and the third group included the remaining three races, which were more pathogenic than the races in the other groups (Fig 1A). Additionally, the accessions were clustered into three major groups (i.e., indicated in green, red, and blue in Fig 1A) based on the LL values for the six *Xoo* races. The accessions in the red group mainly consisted of *indica*-*I* accessions, and were equally resistant to race P1. The resistance level of each accession was based on the average LL for ten plants (two replicates) as follows: LL < 5 cm, 5 cm ≤ LL < 10 cm, 10 cm ≤ LL < 15 cm, and LL ≥ 15 cm represented resistant, moderately resistant, moderately susceptible, and susceptible levels, respectively. A considerable proportion (i.e., from 94.8% for P6 to 98.2% for P3c) of accessions were moderately susceptible or susceptible to *Xoo* races P6, P3c, C5, and GV, respectively (Fig 1B). However, there were similarly high proportions of accessions that were resistant and susceptible to both P1 and P9a, suggesting the *R* genes with large effect on P1 and P9a resistance were carried by only some accessions. The wide range of LLs observed in different rice germplasm accessions indicated there was substantial genotypic variability associated with resistance to *Xoo* races, especially for P9a and P1 (Fig 1C). For all *Xoo* races (except P9a), the average LL of subgroup *indica*-*I* was significantly smaller than that of the other two subgroups. For P9a, the average LL of subgroup *indica*-*II* was significantly higher than that of subgroups *indica*-*I* and *indica*-*III* (*p* < 0.001 for both; Fig 1C).

### Genome-wide association analysis for *Xoo* resistance in *indica* rice

**Fig 2:**
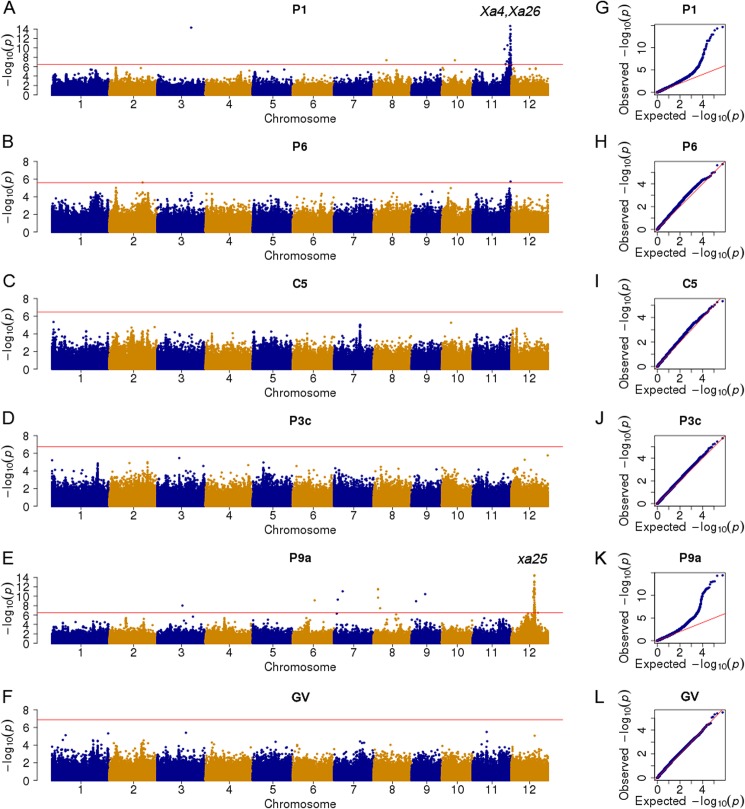
Manhattan and quantile-quantile plots for a genome-wide association study of bacterial blight resistance in *indica* rice. (A, G) P1 (strain PXO61). (B, H) P6 (strain PXO99). (C, I) C5 (strain GD1358). (D, J) P3c (strain PXO340). (E, K) P9a (strain PXO339). (F, L) GV (strain V). The strength of the associations for the lesion lengths caused by six *Xanthomonas oryzae* pv. *oryzae* (*Xoo*) strains is indicated as the negative logarithm of the *p* value for the linear mixed effects model. Based on 1000 permutation tests, the genome-wide significance thresholds (i.e., horizontal red lines in all Manhattan plots) at a significance level of 0.05 were *P* = 3.49 × 10^−7^, 1.82 × 10^−7^, 2.58 × 10^−6^, 3.15 × 10^−7^, 3.50 × 10^−7^, and 1.34 × 10^−7^ for P1, P3c, P6, P9a, C5, and GV, respectively.

We used association mapping to identify genome-wide associated loci underlying the resistance to six *Xoo* races. A total of 279,266 SNPs with a MAF > 0.05 and MDR < 0.2 in the whole population and subpopulation (i.e., whole population without the admixed accessions highlighted in S1B Fig) were used for the association analyses with a linear mixed effects model in the EMMAX program [33]. We constructed the empirical kinship (K) matrix using the BN matrix with 211,943 SNPs (MAF > 0.01 and MDR < 0.05), and covariates (Q) were generated based on the PCA of the population structure. The similarities in the Manhattan plots and quantile-quantile plots for the whole population (Fig 2) and the subpopulation (S2 Fig) suggested the Q + K model effectively controlled the *P*-value inflation and the influence of the population structure on our GWAS. Furthermore, we focused on the GWAS results for the whole population, and detected 121 significantly associated SNPs on chromosomes 2, 3, and 6–12. There were 51, 68, and two SNPs associated with resistance to *Xoo* races P1, P9a, and P6 (Fig 2 and S2 Table), explaining 55.3%, 59.9%, and 13.3% of the variation in LL, respectively. Significantly associated SNPs were not identified for bacterial races P3c, C5, and GV. The 121 detected SNPs were distributed in 96 annotated genes as follows: 60 in intergenic regions, 14 in introns, one in a promoter, 13 in missense variants, 27 in synonymous variants, two in 5′ untranslated regions, two in 3′ untranslated regions, and two in stop codons (S2 Table). Of the 121 significant signals, only SNP rs11_ 28436056 was detected for both P1 and P6. This SNP was located in a *Xa26* family gene (*LOC_Os11g47290*), which encodes a receptor kinase. Gene ontology analysis indicated that these 96 genes were significantly enriched for defense responses, programmed cell death biological processes, and purine ribonucleotide-binding–related functions (S3 Table).

### Regions strongly associated with bacterial blight resistance

**Fig 3:**
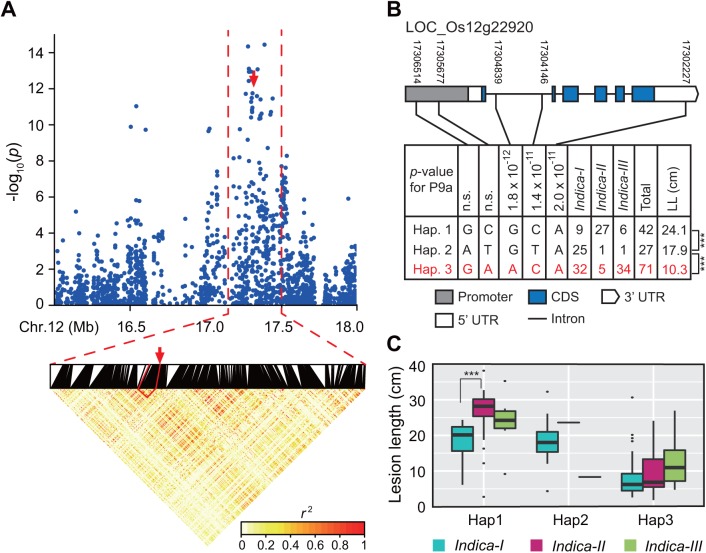
Hotspot region for the resistance to *Xanthomonas oryzae* pv. *oryzae* race P9a and haplotype analysis of the peak associated with the gene on chromosome 12. (A) Local Manhattan plot (top) (16.5–17.5 Mb) and linkage disequilibrium heatmap (bottom) (17.2–17.5 Mb) surrounding the hotspot region on chromosome 12. The arrow indicates the position of the peak single nucleotide polymorphism (SNP) located in *xa25* (*LOC*_*Os12g29220*). Dashed lines indicate the *xa25* region. (B) Gene structure and haplotype analysis of *xa25* in 140 accessions based on five significant SNPs in *xa25*. Haplotypes with fewer than five accessions are not shown. (C) Lesion lengths caused by P9a infections of accessions in three haplotypes of *xa25* in different *indica* subgroups. Box edges represent the 0.25 and 0.75 quantiles with median values indicated by bold lines. Whiskers extend to data no more than 1.5-times the interquartile range, and the remaining data are represented by dots. ‘***’ refers to a significant difference based on Duncan’s multiple comparison tests (*p* < 0.001).

**Fig 4:**
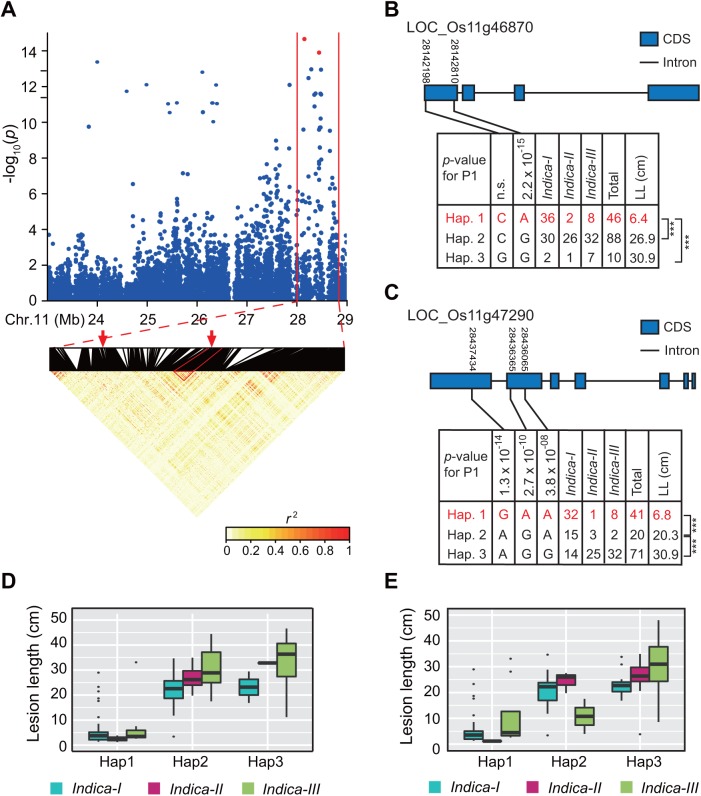
Hotspot region for the resistance to *Xanthomonas oryzae* pv. *oryzae* race P1 and haplotype analysis of the peak associated with the gene on chromosome 11. (A) Local Manhattan plot (top) (23–29 Mb) and linkage disequilibrium heatmap (bottom) (28.0–28.8 Mb) surrounding the hotspot region on chromosome 11. Red arrows and points indicate the positions of the peak single nucleotide polymorphisms located in the *Xa4* candidate gene (i.e., *LOC*_*Os11g46870*) and *Xa26* paralog (i.e., *LOC*_*Os11g47290*), respectively. Dashed lines indicate the *xa25* region. (B) Gene structure and haplotype analysis of the *Xa4* candidate gene (i.e., *LOC*_*Os11g46870*). (C) Gene structure and haplotype analysis of the *Xa26* paralog (i.e., *LOC*_*Os11g47290*). Lesion lengths caused by P1 infections of accessions in three haplotypes of *LOC*_*Os11g46870* (D) and *LOC*_*Os11g47290* (E) in different *indica* subgroups. ‘***’ refers to a significant difference based on Duncan’s multiple comparison tests (*p* < 0.001).

The physical distance between neighboring SNPs was 1–131,763 bp (average: 1349 bp) in our GWAS panel. Thus, we combined adjacent significantly associated SNPs within an LD block (i.e., multiple SNPs exceeding the significance threshold in a 5-Mb region were clustered using an LD *r*^2^ value ≥ 0.25) as a resistance region. Additionally, the SNPs with the lowest *p* values in the LD block were considered the lead SNPs. We detected 12 strongly associated regions containing 120 significant SNPs (including one common SNP for P1 and P6) for three of six *Xoo* races (Table 1). Among these, one hotspot region was associated with resistance to P9a, and spanned an approximately 1.0-Mb interval (i.e., 16,502,066–17,531,046 bp) on chromosome 12, which included 58 (85.3%) of 68 significantly associated SNPs (Fig 3A). Nine significant SNPs (lead SNP rs12_17304839, *P* = 1.8 × 10^−12^) in this region overlapped with *xa25* (*LOC_Os12g29220*) (S2 Table), which is a recessive *R* gene that is responsible for race-specific resistance to P9a in rice [17]. Another hotspot region (i.e., L11), which included 48 significant SNPs associated with resistance to P1, was located at the end of chromosome 11. We determined that 43 (89.6%) of the 48 SNPs in L11 were located in an approximately 1.45-Mb interval (i.e., 27,252,984–28,704,769 bp) (Fig 4A). The corresponding region contained 33 genes that encoded proteins mainly involved in defense responses (e.g., NB-ARC domain-containing protein and protein kinase) according to the Nipponbare reference genome. This region carried the well-known *R* genes *Xa4* and *Xa26* [16, 41]. The lead SNP (i.e., rs11_28142810, *P* = 2.2 × 10^−15^) of region L11 was located in the coding sequence (CDS) of *LOC_Os11g46870*, which encodes a protein kinase. This location corresponded to the *Xa4* gene region based on the physical position of the BAC clone 3H8 that carries *Xa4* [41]. The second leading SNP (i.e., rs11_28437434, *P* = 1.3 × 10^−14^) of region L11 was located in the CDS of *LOC_Os11g47290* (i.e., encoding a receptor kinase), which is the *Xa26* paralog *MRKc* [42] (S2 Table).

**Table 1:** Twelve regions with signals significantly associated with bacterial blight resistance based on a genome-wide association study involving *indica* rice.

Locus ID	Chromosome	Lead SNP position (bp)	LD block	No. of significant SNP in LD block	*Xoo* races	Most significant *p* value	MSU ID of genes harboring lead SNP	Known *R* genes
L1	2	24824096	24824096	1	P6	2.4E-06	LOC_Os02g41450-LOC_Os02g41460	
L2	3	18869855	18869855	1	P9a	9.8E-09	LOC_Os03g33000-LOC_Os03g33010	
L3	3	25435285	25435285	1	P1	4.8E-15	LOC_Os03g45050-LOC_Os03g45070	
L4	6	16516767	16516767	1	P9a	7.5E-10	LOC_Os06g28980	
L5	7	6376395	2727846–6376395	2	P9a	9.0E-12	LOC_Os07g11560-LOC_Os07g11580	
L6	8	3317231	3317110–4873998	4	P9a	3.3E-12	LOC_Os08g06070	
L7	8	9671192	9671192	1	P1	4.2E-08	LOC_Os08g15880	
L8	9	3480616	3480616	1	P9a	1.1E-09	LOC_Os09g07120-LOC_Os09g07130	
L9	9	10293130	10293130	1	P9a	3.6E-11	LOC_Os09g16850-LOC_Os09g16860	
L10	10	9711424	9711424	1	P1	4.3E-08	LOC_Os10g19050-LOC_Os10g19064	
L11	11	28142810	23821163–28704769	48	P1,P6	2.2E-15	LOC_Os11g46870	*Xa4*, *Xa26*
L12	12	17383901	16502066–17531046	58	P9a	3.7E-15	LOC_Os12g29320-LOC_Os12g29330	*xa25*

The other 10 regions (i.e., L1–10) on chromosomes 2, 3, and 6–10 were not associated with previously characterized bacterial blight resistance loci (Table 1, Fig 2 and S2 Table), and represented potentially novel bacterial blight resistance loci. Interestingly, the lead SNPs of six regions (i.e., L2–4 and L8–10) were located in the CDS or intragenic region of genes encoding retrotransposon proteins (i.e., *LOC_Os03g33000*, *LOC_Os03g33010*, *LOC_Os03g45050*, *LOC_Os03g45070*, *LOC_Os06g28980*, *LOC_Os09g07130*, *LOC_Os09g16860*, and *LOC_Os10g19064*) (S2 Table and Table 1). Another distinct peak comprising four SNPs (lead SNP rs8_3317231, *P* = 3.3 × 10^−12^) in region L6 located on chromosome 8 was significantly associated with resistance to P9a. This region contained the *EARLY FLOWERING 7* gene (*LOC_Os08g06070*) (Table 1, Fig 2 and S2 Table). Similarly, we detected one distinct P1 association locus (i.e., L8) on chromosome 8 with a lead SNP (rs8_9671192, *P* = 4.2 × 10^−8^) in the CDS region of the NBS-LRR (Nucleotide binding site-leucine-rich repeats) disease resistance protein gene (*LOC_Os08g15880*). However, we did not identify bacterial blight resistance loci in this region.

### Haplotype analysis for the known and putative novel resistance genes in the *indica* panel

#### Haplotype analysis for the P9a-specific resistance gene *xa25*

The *xa25* haplotypes were built based on two SNPs in a 2-kb region upstream of the *LOC_Os12g29220* promoter, two significantly associated SNPs in the intron region, and one SNP in the 3′ untranslated region (Fig 3B). We detected three haplotypes shared by at least five accessions in 140 of 172 accessions (Fig 3B and S1 Table). Additionally, 45.5% of the variation in LL caused by P9a was explained by *xa25* haplotypes. Multiple comparison tests of the lesions induced by P9a revealed that the average LL of Hap3 was significantly shorter than those of Hap1 and Hap2 (Fig 3B and 3C). The *xa25* gene is a recessive *R* gene that provides rice with race-specific resistance to P9a [17]. It encodes a sucrose transporter (OsSWEET13). Additionally, variations in the *OsSWEET13* promoter sequence results in cryptic recessive resistance to PthXo2-dependent *Xoo* in *japonica* rice [43]. PthXo2-containing strains induce *OsSWEET13* expression in *indica* rice line IR24 because of the presence of an uncharacterized effector-binding site absent in the alleles of the *japonica* rice varieties Nipponbare and Kitaake [43]. We detected Hap3 in the Nipponbare reference genome. Among the 140 accessions with detected haplotypes, 42 (87.5%) of 48 accessions with resistant and moderately resistant reactions to P9a belonged to the Hap3 group (S1 Table). Only one of the 42 Hap1 accessions (i.e., accession ZAO SHOU 691–11) and one of the 27 Hap2 accessions (i.e., accession H 15-23-DA) were resistant to P9a (S1 Table). Additionally, 92.6% of Hap2 accessions belonged to the *indica-I* subgroup, while 64.3% of Hap1 accessions belonged to the *indica-II* subgroup. Meanwhile, 45.1% and 47.9% of the Hap3 accessions belonged to the *indica-I* and *indica-III* subgroups, respectively. These results explained the phenotypic variability among the three *indica* subgroups in response to *Xoo* race P9a (Fig 1C).

#### Haplotype analysis for the *Xa26* paralog *MRKc*

The *R* gene *Xa26* provides resistance to *Xoo* race P1 [16]. The top hit for *Xa26* following a blastp search of the Nipponbare reference genome (IRGSP 1.0) was *LOC*_*Os11g47210*, which encodes a receptor kinase-like protein. We did not detect any significant association signals or a resistant haplotype associated with *LOC*_*Os11g47210* (data not shown). However, we identified one strong association signal for P1 located at around 28,437,434 bp (i.e., rs11_28437434, *P* = 1.3 × 10^−14^) on chromosome 11. There were also five significant SNPs for P1 and one significant SNP for P6 located in the CDS region of *LOC*_*Os11g47290* (encoding a receptor kinase), which is a *Xa26* paralog (i.e., *MRKc*) [42] (Fig 4A and S2 Table). We mainly analyzed the *LOC*_*Os11g47290* haplotype, and identified three major haplotypes shared by at least five accessions in 132 accessions based on three SNPs with strong association signals in the gene coding region (Fig 4C and S1 Table). These haplotypes explained 45.3% and 32.2% of the phenotypic variations in the lesions induced by P1 and P6, respectively. The lesions induced by P1 in Hap1 accessions were significantly shorter than those of the Hap2 and Hap3 accessions (*p* < 0.001 for both; Fig 4C and 4E). Additionally, 29 (72.5%) of 40 accessions with resistant reactions to P1 belonged to the Hap1 group (Fig 4C and S1 Table). Only two of the 20 Hap2 accessions (i.e., INIAP 415 and HAO HOM) and one of the 71 Hap3 accessions (i.e., TAICHUNG NATIVE 1) were resistant to P1 (S1 Table). Meanwhile, 52.5%, 86.2%, and 76.2% of the Hap1, Hap2, and Hap3 accessions belonged to *indica-I*, *indica-III*, and *indica-III* subgroups, respectively. These results explained the phenotypic variability among the three *indica* subgroups in response to *Xoo* race P1 (Fig 1C).

#### Haplotype analysis for a *Xa4* candidate gene

The *Xa4* gene has been reported to provide resistance against *Xoo* race P1 [1]. A comparison between the physical positions of BAC clones 3H8 and X4-88 carrying *Xa4* [41] and the significantly associated SNPs in this study suggested a protein kinase gene (i.e., *LOC*_*Os11g46870*) might be a *Xa4* candidate, in which the strongest association (i.e., rs11_28142810, *P* = 2.2 × 10^−15^) for P1 resistance was detected in the coding region (Fig 2 and S2 Table). Three major haplotypes shared by at least five accessions were detected in 146 accessions based on two SNPs in the coding region of *LOC*_*Os11g46870* (Fig 4A). We determined that 54.5% and 30.5% of the phenotypic variability in lesions induced by P1 and P6 were explained by *LOC*_*Os11g46870* haplotypes, respectively. A comparison of the LLs for the three haplotypes following an infection by *Xoo* race P1 revealed that Hap1 accessions had significantly shorter LLs than the other two haplotypes (*p* < 0.001 for both; Fig 4B and 4D). Furthermore, 33 (82.5%) of 40 accessions with a resistant reaction to P1 infection belonged to the Hap1 group, and 26 accessions belonged to the *indica-I* subgroup (Fig 4B and S1 Table). In contrast, only one of the 88 Hap2 accessions (i.e., INIAP 415) and 10 of the Hap3 accessions were resistant to P1 (S1 Table). Additionally, 78.2% and 70.0% of the Hap1 and Hap3 accessions belonged to the *indica-I* and *indica-III* subgroups, respectively, whereas the Hap2 accessions were relatively evenly distributed among the *indica-I*, *indica-II*, and *indica-III* subgroups. These results explained the phenotypic variations observed among the three *indica* subgroups in response to *Xoo* race P1 (Fig 1C).

## Discussion

### Distinct differences in bacterial blight resistance among *indica* rice subgroups

We conducted a GWAS involving 172 global *indica* accessions infected by representative strains of six *Xoo* races from China and the Philippines to analyze the genetic basis of bacterial blight resistance in rice, and detected two hotspot regions associated with *Xoo* resistance. One hotspot region consisted of an approximately 1.45-Mb interval (i.e., 27,252,984–28,704,769 bp) on chromosome 11. This region included 89.6% (i.e., 43 out of the 48) of the significant SNPs associated with resistance to P1. The other identified hotspot region spanned an approximately 1.0-Mb interval (i.e., 16,502,066–17,531,046 bp) on chromosome 12. This region contained 85.3% (i.e., 58 out of 68) of the significant SNPs associated with resistance to P9a (Fig 2 and S2 Table). Our results suggest that chromosomes 11 and 12 were important for the evolution of rice disease resistance. At the genome level, loci associated with bacterial blight resistance exhibited race specificity, similar to some reported *R* genes and QTLs [1, 24, 25]. In this study, only two SNPs with weak but significant signals associated to resistance for P6 were detected (Table 1 and Fig 2), no any SNPs significantly associated resistance for *Xoo* races P3c, C5, and GV was identified in the tested rice accessions due to limited phenotypic variation (Fig 1), suggesting lack of *R* gene/QTL with large effect on resistance for these races in the association panel. It is generally accepted that GWAS has low power to find associations for rare alleles [44]. To clarify whether low allele frequency resulted in false positive associations in GWAS, further studies will be needed to ascertain the functions of resistance candidate genes by expression profiling or genetic transformation.

Our GWAS and haplotype results provided some interesting information on the differentiation and accumulation of resistance loci for *Xoo* in rice. Bacterial blight is prevalent in both tropical and temperate areas, but outbreaks and epidemics of this disease frequently occur in the tropics because of a favorable climate for the survival, propagation, and infection of *Xoo* [1, 45]. During the co-evolution of plant hosts and pathogens, the strong selection pressure resulting from diverse pathogen populations might have induced *indica* rice lines to generate novel disease resistance specificities or evolve novel effectors to overcome the effects of genes associated with disease susceptibility. Considerable selection pressures from various *Xoo* races have contributed to the diversification of disease resistance loci in *indica*. This has resulted in the differentiation of bacterial blight resistance among the *indica* subgroups. In this study, 45.1% and 47.9% of the *xa25* haplotype accessions resistant to P9a belonged to *indica-I* and *indica-III* subgroups, respectively (Fig 3B and S1 Table). However, 92.6% of the susceptible *xa25* haplotype accessions (i.e., Hap2) belonged to the *indica-I* subgroup, in which 13 accessions resistant to P1 carried *LOC*_*Os11g46870* or *LOC*_*Os11g47290* resistance haplotypes (Fig 4 and S1 Table). Nipponbare reference genome indicated that this *japonica* rice variety carries *xa25* [43] as well as the associated disease resistance haplotype. These results suggested that the effects of the *xa25* alleles in *indica* accessions were overcome by P9a during the co-evolution between the host and pathogen.

Breeding for bacterial blight resistance has been one of the most important objectives among rice breeders in Asia since the 1970s. Rice germplasm carrying *Xa4* and *Xa3/Xa26* exhibit a broad resistance to Chinese *Xoo* races, and have been obtained from the International Rice Research Institute and used as the bacterial blight resistance donor in rice breeding programs [3]. The results in study suggest that breeding for bacterial blight resistance involved the end of the long arm of chromosome 11 (Fig 2 and Table 1), which carries several bacterial blight resistance genes (e.g., *Xa4*, and *Xa26*). The haplotype analysis indicated that accessions with P1 resistance haplotypes related to the *Xa4* candidate gene (i.e., *LOC*_*Os11g46870*), *Xa26* paralog gene (i.e., *LOC*_*Os11g47290*), and a P9a-specific susceptible haplotype of the *xa25* gene (i.e., *LOC*_*Os12g29220*) were associated with differential disease susceptibilities among the *indica* subgroups (Fig 3 and Fig 4). The P1 resistance haplotypes associated with the *Xa4* candidate gene (i.e., *LOC*_*Os11g46870*) and *Xa26* paralog gene (i.e., *LOC*_*Os11g47290*) were more frequently detected in the *indica-I* subgroup than in the *indica-II* and *indica-III* subgroups (Fig 4B and 4C). This implies that conventional breeding has applied a strong selection pressure for certain *R* genes, and the breeding process for disease resistance of certain varieties has contributed to the accumulation of BB resistance loci.

### Putative roles of transposable elements affecting bacterial blight resistance in rice

We identified several retrotransposons and transposons among the candidate genes anchored by the SNPs associated with bacterial blight resistance, including significant SNPs (S2 Table). Transposable elements have been suggested to contribute to the evolution of genes by providing *cis*-regulatory elements leading to changes in expression patterns [46–49]. Stress-induced changes to retrotransposons may play a role in generating host genetic plasticity in response to environmental stresses [50–52]. In rice, *Xa21* encodes a receptor-like kinase and confers resistance to *Xoo*. This gene is a member of a multi-gene family in which 17 transposon-like elements have been identified in the 5′ and 3′ flanking regions and introns [49, 53, 54]. Researchers have attempted to diversify the rice bacterial blight resistance genes. Efforts have involved point mutations [55], deletion and duplication of intragenic DNA repeats encoding blocks of leucine-rich elements subject to adaptive selection [56], intragenic and extragenic recombinations [57], and insertion of transposable elements [49]. Our results suggest that transposable elements have highly enriched the diversity of bacterial blight resistance genes during evolution. Additionally, a large-scale attempt to identify and annotate loci provides useful insights into the genetic control of *indica* rice traits.

### Candidates for fine-mapped genes and novel loci

We identified 12 bacterial blight resistance loci containing 121 significantly associated signals using 317,894 SNPs. Two hotspot regions (i.e., L11 and L12) were located within or nearby cloned *R* genes (i.e., *xa25* and *Xa26*) and a fine-mapped *R* gene (i.e., *Xa4*). These observations revealed the relatively high resolution of a GWAS involving a relatively large population, which increased our ability to investigate genetic diversity and a high-density SNP map. Additionally, our haplotype analysis results suggest that the *Xa4* candidate gene (i.e., *LOC*_*Os11g46870*) and the *Xa26* paralog (i.e., *LOC*_*Os11g47290*) likely confer resistance to P1. Moreover, two novel loci on chromosomes 8 [i.e., L6 (rs8_3317231, *P* = 3.3 × 10^−12^) and L7 (rs8_9671192, *P* = 4.2 × 10^−8^)] were significantly associated with resistance to P9a and P1, respectively. These results provide valuable information for future studies involving bacterial blight resistance gene cloning. Moreover, the identified diverse rice accessions carrying fine-mapped and novel loci from nearly 20 countries will provide more available donors in breeding programs aiming at developing bacterial blight resistance in different rice growing regions.

## Conclusions

Our study provides new insights into the genetic basis of the evolution of bacterial blight resistance in rice. The findings reported herein may be useful for knowledge-based crop improvement. Future research will focus on validating the effects of these candidate genes and their functional variants. We will use genetic transformations and DNA insertion mutant screens to verify that these genes confer bacterial blight resistance to rice.

## Supporting information

S1 Fig Population structure of the 172 *indica* rice accessions. (A) Neighbor-joining tree of 172 accessions. (B) Principal component analysis plots for the first two components of 172 accessions. (C) Distribution of the estimated subpopulation components for each accession as determined by ADMIXTURE.
(PDF)

S2 Fig Manhattan and quantile-quantile plots of a genome-wide association study of bacterial blight resistance in *indica* rice subpopulations without admixed accessions based on principal component analysis. (A, G) P1 (strain PXO61). (B, H) P6 (strain PXO99). (C, I) C5 (strain GD1358). (D, J) P3c (strain PXO340). (E, K) P9a (strain PXO339). (F, L) GV (strain V). The strength of the associations for the lesion lengths caused by representative strains of six *Xanthomonas oryzae* pv. *oryzae* (*Xoo*) races is indicated as the negative logarithm of the *p* value for the linear mixed effects model.
(PDF)

S1 Table Information regarding the 172 analyzed *indica* accessions. (XLSX)

S2 Table All 121 significant association signals for three *Xanthomonas oryzae* pv. *oryzae* races. (XLSX)

S3 Table Gene ontology enrichment results for the annotated genes carrying significant single nucleotide polymorphisms associated with bacterial blight resistance based on a genome-wide association study. (XLSX)
