# Supplementary figures and images for: Genome-wide association analysis identifies resistance loci for bacterial blight in a diverse collection of indica rice germplasm
Source: PLoS One. 2017 Mar 29;12(3):e0174598. doi: 10.1371/journal.pone.0174598 (PMC5371361; doi:10.1371/journal.pone.0174598)

**A**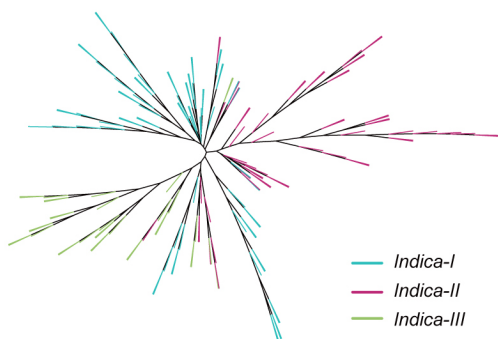**B**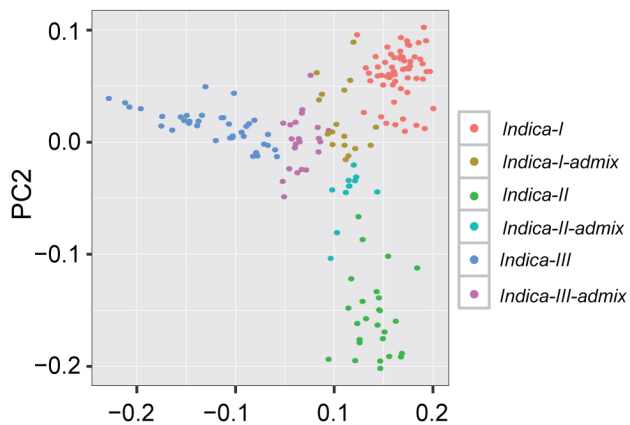**C**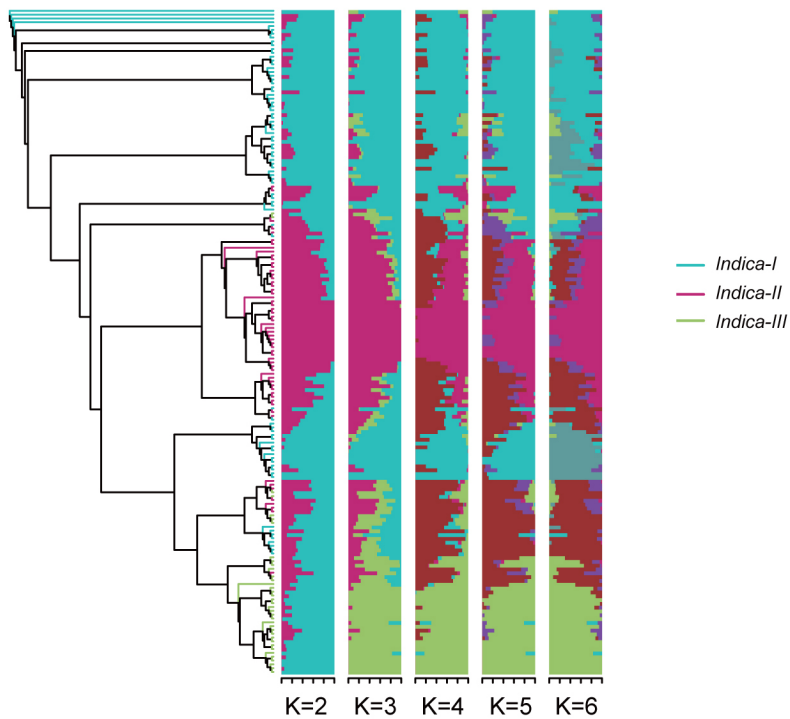

Supplement: S1 Fig — (A) Neighbor-joining tree of 172 accessions. (B) Principal component analysis plots for the first two components of 172 accessions. (C) Distribution of the estimated subpopulation components for each accession as determined by ADMIXTURE. (PDF) [file pone.0174598.s001.pdf]

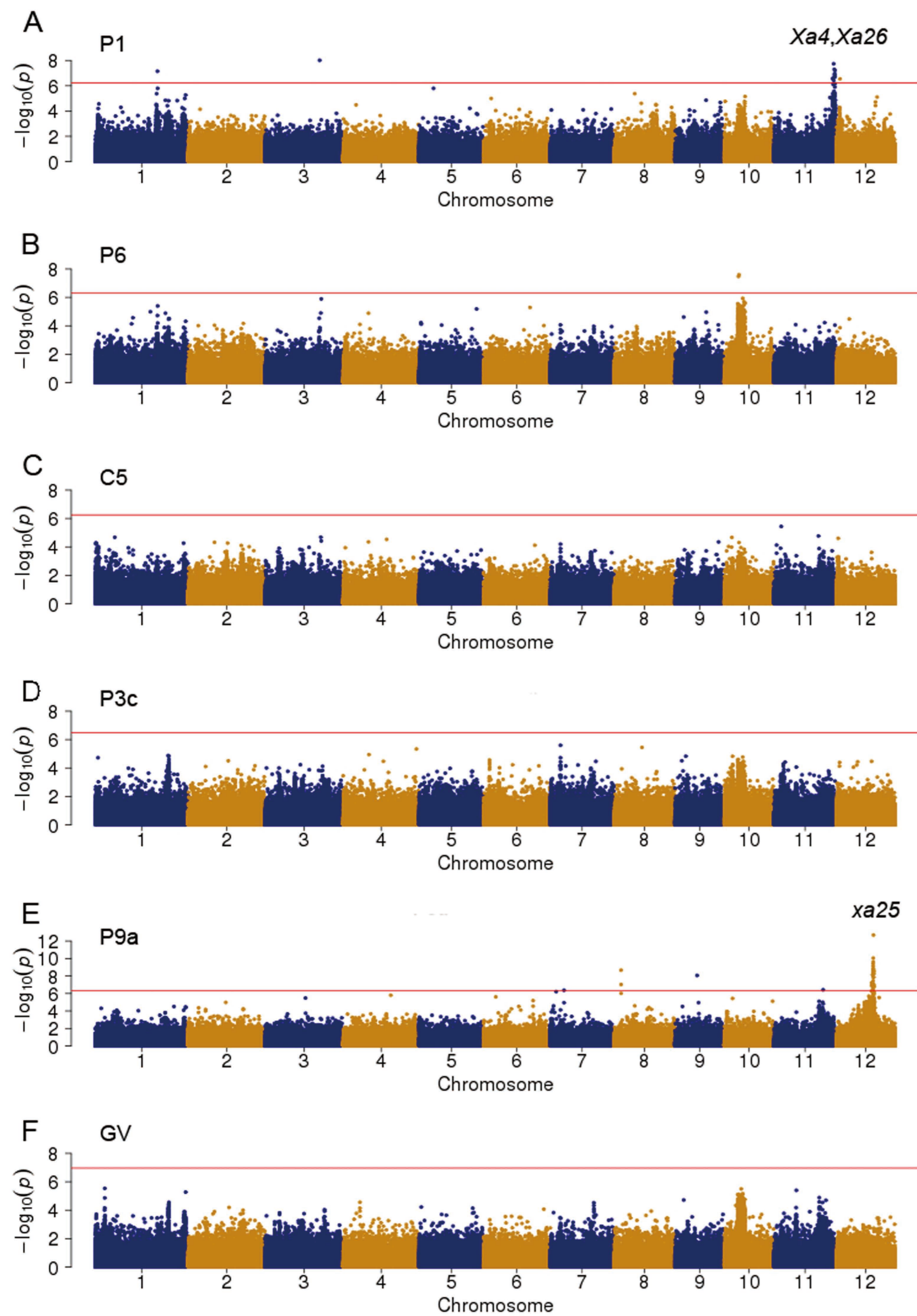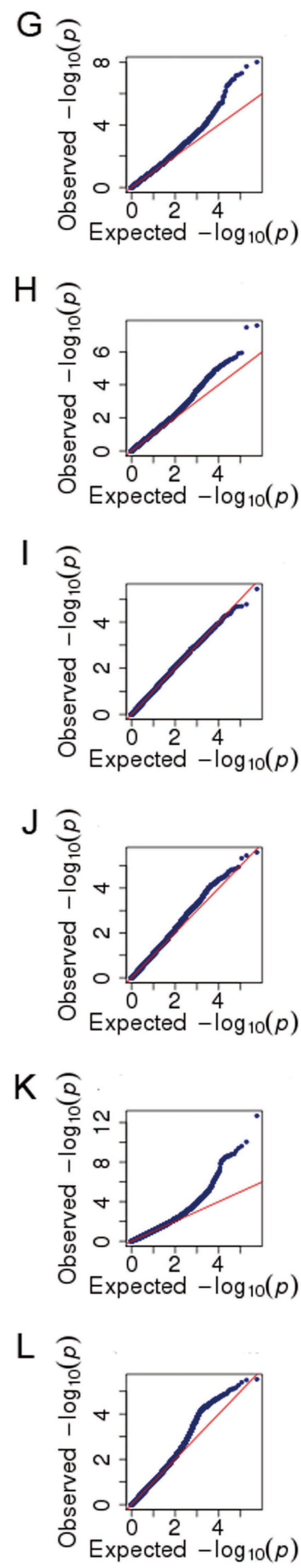

Supplement: S2 Fig — (A, G) P1 (strain PXO61). (B, H) P6 (strain PXO99). (C, I) C5 (strain GD1358). (D, J) P3c (strain PXO340). (E, K) P9a (strain PXO339). (F, L) GV (strain V). The strength of the associations for the lesion lengths caused by representative strains of six Xanthomonas oryzae pv. oryzae (Xoo) races is indicated as the negative logarithm of the p value for the linear mixed effects model. (PDF) [file pone.0174598.s002.pdf]
